# Altered Functional Hubs and Connectivity in Type 2 Diabetes Mellitus Without Mild Cognitive Impairment

**DOI:** 10.3389/fneur.2020.01016

**Published:** 2020-09-11

**Authors:** Yifan Li, Yi Liang, Xin Tan, Yuna Chen, Jinquan Yang, Hui Zeng, Chunhong Qin, Yue Feng, Xiaomeng Ma, Shijun Qiu

**Affiliations:** ^1^First Clinical Medical College, Guangzhou University of Chinese Medicine, Guangzhou, China; ^2^Department of Radiology, The First Affiliated Hospital of Guangzhou University of Chinese Medicine, Guangzhou, China; ^3^Department of Radiology, The First Affiliated Hospital of Guangdong Pharmaceutical University, Guangzhou, China

**Keywords:** type 2 diabetes mellitus, resting state, functional connectivity, degree centrality, cognitive function

## Abstract

**Background:** Type 2 diabetes mellitus (T2DM)-related cognitive decline is associated with neuroimaging changes. However, only a few studies have focused on early functional alteration in T2DM prior to mild cognitive impairment (MCI). This study aimed to investigate the early changes of global connectivity patterns in T2DM by using a resting-state functional magnetic resonance imaging (rs-fMRI) technique.

**Methods:** Thirty-four T2DM subjects and 38 age-, sex-, and education-matched healthy controls (HCs) underwent rs-fMRI in a 3T MRI scanner. Degree centrality (DC) was used to identify the functional hubs of the whole brain in T2DM without MCI. Then the functional connectivity (FC) between hubs and the rest of the brain was assessed by using the hub-based approach.

**Results:** Compared with HCs, T2DM subjects showed increased DC in the right cerebellum lobules III–V. Hub-based FC analysis found that the right cerebellum lobules III–V of T2DM subjects had increased FC with the right cerebellum crus II and lobule VI, the right temporal inferior/middle gyrus, and the right hippocampus.

**Conclusions:** Increased DC in the right cerebellum regions III–V, as well as increased FC within cerebellar regions and ipsilateral cerebrocerebellar regions, may indicate an important pathophysiological mechanism for compensation in T2DM without MCI.

## Introduction

Type 2 diabetes mellitus (T2DM) is a systemic metabolic disease, resulting in severe complications of multiple systems. Recently, T2DM-related cognitive dysfunction has been paid more attention (1–3). Mild cognitive impairment (MCI), an important complication of T2DM, is intermediate transition state between age-appropriate cognition and dementia (4). Although the integral cognitive function kept “normal” up for a long time before MCI, there has been a gradually imperceptible decline in some aspects of cognitive function (5, 6). Early detection of the brain functional changes of this stage could be clinically significant, because it might provide theoretical evidence for explaining cognitive changes related to T2DM and might greatly postpone the onset of MCI.

Currently, neuroimaging methods have been generally applied to probe the mechanism of T2DM-related cognitive impairment (7–10). However, only a few studies kept a watchful eye on the early neuroimaging changes in T2DM subjects without MCI, and the underlying mechanisms have not been entirely elaborated. In a previous study, neurovascular decoupling pattern in T2DM without MCI was assessed using a variety of imaging methods (5). In another study, alterations in connectivity within the Papez circuit in T2DM patients without MCI were identified, and the relationships between these alterations and insulin resistance have been determined (11). However, there were no comprehensive and sensitive assessments on cognitive function, or subjects in these studies were older. Sun et al. found that functional connectivity (FC) between the hippocampus and certain brain structures had decreased significantly in T2DM subjects without MCI (12). However, results of the region of interest (ROI)-based FC analysis should be interpreted in caution, as the selected ROIs are mainly based on the prior knowledge, leading to different and unpredictable findings (13). Our previous studies showed microstructure of the white matter changes in T2DM without MCI in several brain regions including the bilateral cingulum lobe and thalami (14), pons, and left temporal pole (15). It is believed that alterations in brain structure may lead to changes in brain function (16–19). Abnormalities in FC are largely related to cognitive abnormalities (20). Therefore, comprehensive whole-brain FC analyses were needed.

Another neuroimaging method, the degree centrality (DC), is used to explore the distribution of the local FC by using resting-state functional magnetic resonance imaging (rs-fMRI) technique (21–23). It does not need to pick out seed points, nor does it need to consider the relationships between brain regions. It can investigate the overall performances and patterns of functional connectome with higher speed than traditional methods, and it can perform whole-brain analysis at the voxel level to eliminate biases caused by selection of brain regions based on prior assumptions (22, 23).

DC has been widely used to quantify node features of networks, and increased DC regions are defined as functional hubs (22). Previous researches using DC analysis showed altered brain functional hubs and connectivity in T2DM subjects (5, 23, 24). However, subjects enrolled in these studies had clinical cognitive impairment or lacked cognitive assessment. In the present work, we aimed to explore the early neurological changes of T2DM without MCI based on DC. Specifically, DC was firstly performed to evaluate the altered brain functional hubs of T2DM patients compared with the control group; then a hub-based analysis of the interactions among these functional hubs or FC between hubs and the rest of brain was conducted to further explore internal changes of connections. A large number of cognitive scales were used to assess the cognitive function of T2DM subjects as a whole.

## Materials and Methods

### Subjects

All the T2DM subjects were enrolled from inpatients and outpatients of the First Affiliated Hospital of Guangzhou University of Chinese Medicine from November 1, 2017, to December 20, 2019 (Table 1). The diagnostic criteria for T2DM were defined by the American Diabetes Association (ADA). The healthy control (HC) group consisted of healthy people who underwent routine physical examination. All subjects were right-handed and had more than 6 years of education. They were all Han Chinese and native Chinese speakers. None of the subjects had obvious cognitive impairment or dementia, and their Montreal Cognitive Assessment (MoCA, Beijing edition) scores were ≥26. The study was authorized by the Medical Research Ethics Committee of Guangzhou University of Chinese Medicine, and written informed consent was obtained from each participant before the experiment. HC subjects were matched with T2DM subjects in reference to age, sex, and education. Participants associated with neurological and psychiatric diseases (e.g., brain injury and cerebrovascular accident, brain tumors, epilepsy, depression, schizophrenia, and Parkinson's disease), or systematic disease(s), complications associated with diabetes (such as diabetic retinopathy, diabetic nephropathy, and peripheral neuropathy), alcohol dependence or history of drug abuse, other types of diabetes, and contraindications to MRI were excluded. Finally, a total of 38 HC subjects and 34 T2DM subjects were enrolled. All the T2DM subjects enrolled in the current study received initiating oral metformin following insulin via a pump or subcutaneous injection during hospitalization. Their blood sugar levels were well-controlled. None of the T2DM subjects received intranasal insulin.

**Table 1:** Demographics, clinical data, cognitive assessment of T2DM and HCs.

	**Diabetic subjects (*n* = 34)**	**Healthy controls (*n* = 38)**	**Statistics**	***P*-value**
**DEMOGRAPHICS**				
Age (years)	49.41 ± 5.58	47.42 ± 6.81	*t* = 1.347	0.182
Gender (M/F)	22/12	19/19	χ^2^ = 1.58	0.208
Education (years)	9 (6, 16)	10 (6, 16)	*z* = −0.187	0.852
**CLINICAL DATA**				
BMI (kg/m^2^)	25.04 ± 2.40	22.72 ± 2.55	*t* = 3.945	0.000^*^
SBP (mmHg)	133.06 ± 15.64	117.82 ± 11.10	*t* = 4.808	0.000^*^
DBP (mmHg)	85 (66, 120)	76 (71, 110)	*z* = −3.117	0.002^*^
HbA1c (%)	8.73 ± 2.15	N/A	N/A	N/A
FBG (mmol/L)	7.59 (3.66, 17.50)	N/A	N/A	N/A
FINS (μIU/ml)	5.94 (0.79, 18.90)	N/A	N/A	N/A
TG (mmol/L)	1.64 (0.64, 7.61)	N/A	N/A	N/A
TC (mmol/L)	4.51 ± 0.98	N/A	N/A	N/A
LDL (mmol/L)	3.28 ± 1.11	N/A	N/A	N/A
**COGNITIVE ASSESSMENT**				
MoCA score	26 (26, 29)	28 (26, 30)	*z* = −3.174	0.002^*^
AVLT (immediate)	20.00 ± 4.83	21.18 ± 5.48	*t* = −0.968	0.337
AVLT (5 min)	7 (4, 12)	8 (4, 14)	*z* = −1.494	0.135
AVLT (20 min)	7.74. ± 2.47	8.34 ± 2.47	*t* = −1.041	0.302
AVLT (recall)	11.5 (6, 12)	12 (5, 13)	*z* = −1.245	0.213
Grooved pegboard (R)	77.05 (59.3, 181)	73.5 (55, 130.8)	*z* = −1.574	0.115
Grooved pegboard (L)	83 (70, 182)	80.35 (53, 193)	*z* = −1.055	0.291
TMT-A	53.5 (21, 156)	54 (23, 124)	*z* = −0.914	0.361
TMT-B	45.5 (19, 118)	42.8 (23, 89)	*z* = −0.790	0.430
DST (forward)	8 (5, 12)	8 (6, 11)	*z* = −0.536	0.592
DST (inverse)	4 (2, 10)	4 (2, 10)	*z* = −0.442	0.659
CDT	3 (1, 3)	3 (2, 3)	*z* = −1.967	0.049^*^
SDT	42.29 ± 10.28	50.29 ± 14.32	*t* = −2.694	0.009^*^

*Values distributed normally or non-normally are presented as mean ± SD or median (minimum, maximum)*.

*BMI, body mass index; SBP, systolic blood pressure; DBP, diastolic blood pressure; FBG, fasting blood glucose; FINS, fasting insulin; TG, triglyceride; TC, total cholesterol; LDL, low-density lipoprotein; MoCA, Montreal Cognitive Assessment; AVLT, auditory verbal learning test; TMT, trail making test; GPT, grooved pegboard test; SDT, symbol digit test; CDT, the clock drawing test; DST, the digital span test*.

* *P < 0.05*.

### Demographic and Clinical Characteristics

General demographic information and clinical measurement of all the subjects were collected, including age, gender, education level, body mass index (BMI), and blood pressure. The laboratory tests were available for T2DM, including fasting blood glucose (FBG), fasting insulin (FINS), triglyceride (TG), total cholesterol (TC), low-density lipoprotein (LDL), and glycosylated hemoglobin (HbA1c).

### Cognitive Assessment

All subjects received a range of neuropsychological tests, which were sensitive and commonly used in T2DM-related studies, including the MoCA (25), auditory verbal learning test (AVLT) (26), trail making test (TMT; including parts A and B) (27), grooved pegboard test (GPT) (28), symbol digit test (SDT) (29), the clock drawing test (CDT) (30), and the digital span test (DST, including forward and backward) (31). The AVLT involved four parts: immediate recall, short-term delayed recall, long-term delayed recall, and recognition. The whole process took at least 35 min to complete assessment.

### Image Acquisition

MRI data were acquired on a 3-T GE SIGNA clinical MRI scanner with an eight-channel phased-array head coil. Scanning consisted of two parts: conventional sequences were used for screening lesions, and functional sequences were used for experiment processing. First, all participants underwent conventional brain axial T1-weighted, T2-weighted, and fluid-attenuated inversion recovery (FLAIR) images to rule out brain organic diseases and white matter hyperintensity (WMH) lesions (15). Images of conventional sequences (T1, T2, and FLAIR) were inspected, respectively, by two radiologists who had a wealth of work experience, and none of the subjects were excluded after the conventional scans. Functional images were obtained using a gradient-echo planar sequence. The scanning parameters were consistent with our previous research (7).

The scan was performed within 2–3 h after dinner (7–8p.m.), and the time interval between the scanning and the neuropsychological tests should be at least 0.5 h. Needle injection (for blood tests or therapies) or sleeplessness before scanning also was avoided. The blood glucose levels were all well-controlled before scanning. During the scanning, the subjects were asked to avoid systemic thinking or falling asleep.

### Data Preprocessing

The entire calculation process was carried out on the MATLAB R2014a platform. Data analysis tool RESTplus was used to preprocess the original rs-fMRI data (32). The first 10 images were discarded and the remaining 175 time-point images were preprocessed. The main procedure was conducted as follows: (1) DICOM-NIFTI format conversion. (2) Slice timing: the acquisition time of all voxels was kept consistent through rectification, and each time point was aligned to the time point of the most middle layer. (3) Head movement correction: subjects were excluded if the maximum cumulative head motion exceeded 2 mm in translation or 2° in rotation along any direction. (4) Spatial normalization: the brain images of all subjects were aligned to the same standard space to solve the problems of individual differences in brain size and scanning positions. After alignment, statistical comparison based on voxel could be conducted in the Montreal Neurological Institute (MNI) space, and the resampled voxel size was 3 × 3 × 3 mm. (5) Remove linear drift: the trend signal generated during scanning period should be decomposed from the original time series data. (6) Regression covariates: the interference signal should be removed by a linear regression model, which includes (6) head motion parameters, white matter signal, and cerebrospinal fluid signal. (7) Filtering: data were filtered from 0.01 to 0.08 Hz.

The brain intrinsic connectivity network was constructed at the voxel level. The temporal Pearson's correlation of time series and the similarity of rs-fMRI signals between pairs of voxels were measured to calculate a correlation matrix. For a weighted graph, which is more robust against confounding factors, DC is defined as the sum of weights from edges connecting to a node (also sometimes referred to as the node strength) (22).

The DC in RESTplus software was used to calculate the DC value based on correlation thresholds (absolute value of Pearson's *r* > 0.2) to exclude weak correlations, which may be caused by signal noise based on previous studies (22, 33, 34). Then smooth was performed with a 6 × 6 × 6 mm^3^ Gaussian kernel.

The hub-based FC analysis was also conducted by RESTplus. Peak MNI coordinates of the brain functional hubs were acquired by group comparison of DC maps, and the hubs were considered to be the center of ROI with a 6-mm radius. The average time series of ROI was extracted by using the anatomical automatic labeling (AAL) template in the RESTplus software package and correlated with the time series of voxel in other brain regions of the whole brain. And then a Fisher z-transform was used to improve the normality of the correlation coefficients.

### Statistical Analysis

#### Demographic and Clinical Characteristics Analysis

Kolmogorov–Smirnov test (33) was applied to detect the normality of the continuous variables, including the demographic data and biochemical characteristics of the T2DM subjects and HCs. Independent two-sample *t*-tests were used if the data meet normal distribution and variance homogeneity; otherwise, Mann–Whitney non-parametric tests were used. The chi-square test was used to evaluate intergroup difference in gender. All statistical analyses were using the IBM Statistical Package for the Social Sciences 22.0 software (IBM SPSS Inc., Chicago, IL, USA). The significance level was set at *P* < 0.05.

#### Intergroup Comparison of Resting-State Degree Centrality and Hub-Based Functional Connectivity

The two-sample *t*-test in the RESTplus data analysis kit V1.21 software was used to compare the DC statistical parameter charts of the two groups of subjects. The built-in brain mask of RESTplus (61 × 73 × 61 mm) was used as the registration template, and the initial threshold was *P* < 0.01, and the Gaussian random field (GRF) correction was performed. Voxel *P* < 0.01 and cluster *P* < 0.05 were set. The inspection is double tail inspection. The corrected cluster size threshold is 179 voxels. Then, an independent sample *t*-test and a GRF were also used to explore the differences between T2DM subjects and HCs in terms of FC.

These brain regions were positioned, and the location, size, peak MNI coordinates, and *t*-values of statistically significant brain regions were recorded according to the AAL partition template.

#### The Relationship Between Clinical Biochemical Index and Neurocognitive Function

Partial correlation analysis was applied to analyze the correlation between various clinical biochemical indicators (HbA1c, TC, TG, FINS, FBG, and LDL) and scores of different neurocognitive function scales (MoCA, AVLT, TMT-a, TMT-b, GPT, DST, CDT, and SDT) in the T2DM group after controlling for gender, age, and education level. *P* < 0.05 was considered statistically significant.

## Results

### Demographic, Clinical, and Cognitive Characteristics

Demographic, clinical, and cognitive information of all the subjects is listed in Table 1. There was no statistical difference in age, gender, and education levels between the two groups. Compared with HCs, the T2DM group had significantly higher levels of BMI and blood pressure and exhibited worse performance on the tests of MoCA, CDT, and SDT (*P* < 0.05).

### Intergroup Differences in Degree Centrality

**Figure 1:**
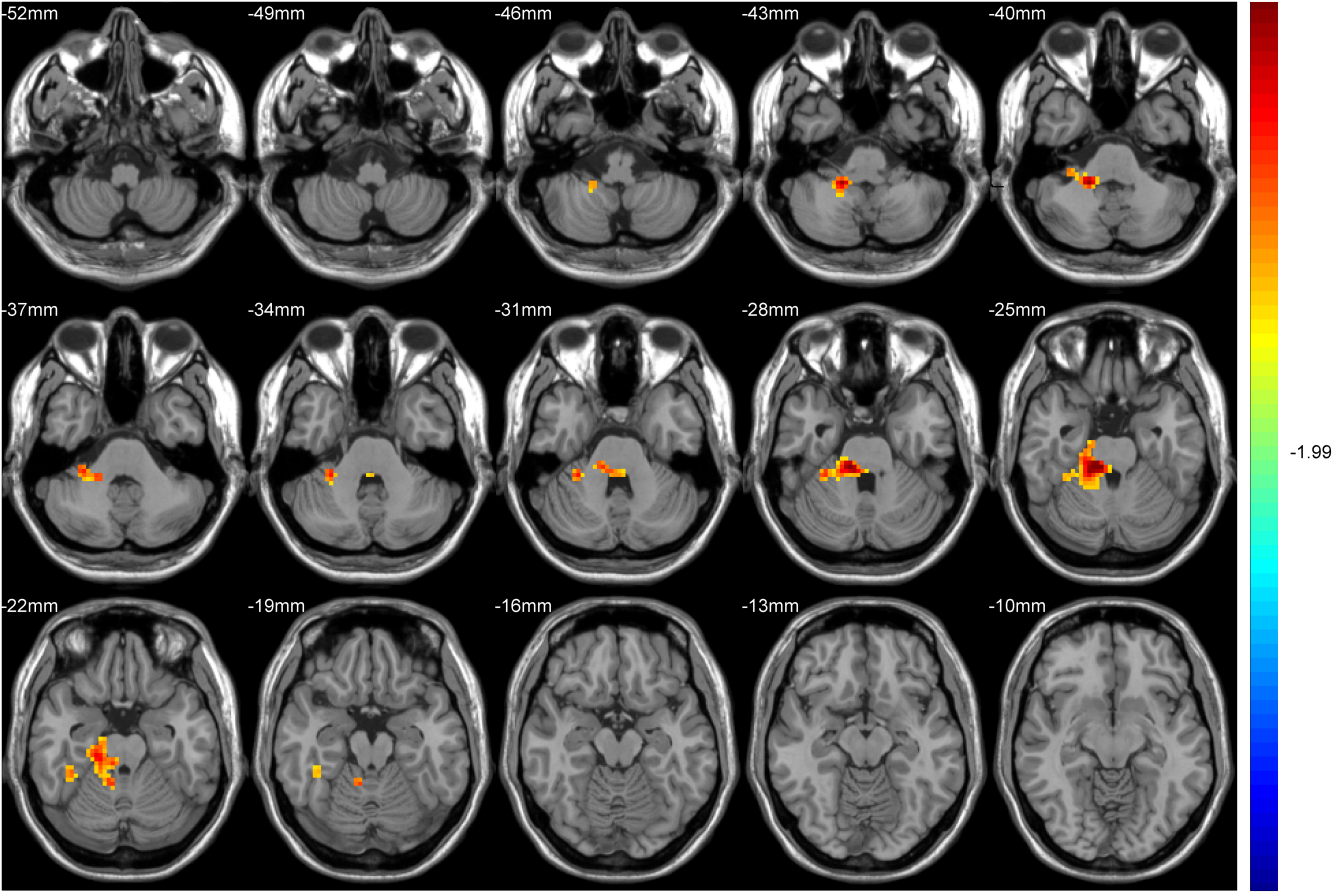
The colored brain regions represent significantly increased degree centrality (DC) in type 2 diabetes mellitus (T2DM) subjects compared with healthy controls (HCs). The color bar indicates the *t*-value from two-sample *t*-tests.

After GRF multiple comparison correction (voxel *P* < 0.01, cluster *P* < 0.05), only one cluster survived in the T2DM group compared with the HC group in the brain areas with increased DC (Table 2, Figure 1), involving the right cerebellum areas III–V. To further verify the validity and reliable of the results by using 0.2 as threshold in the DC analysis, the thresholds of 0.1 and 0.3 had also been processed (Supplementary Figures 1, 2). The location of the peak MNI point and the cluster were consistent, but the size of the cluster was slightly altered.

**Table 2:** Brain regions with increased DC in T2DM compared with HCs.

**Cluster**	**Brain regions**	**Peak MNI**			**Number of voxels**	***t*-value**
		**X**	**Y**	**Z**		
	Right cerebellum III Right cerebellum IV–V	12	−33	−24	183	4.9013

*DC, degree centrality; MNI, Montreal Neurological Institute; X, Y, Z, coordinates of primary peak locations in the MNI space*.

### Intergroup Differences in Functional Connectivity

**Figure 2:**
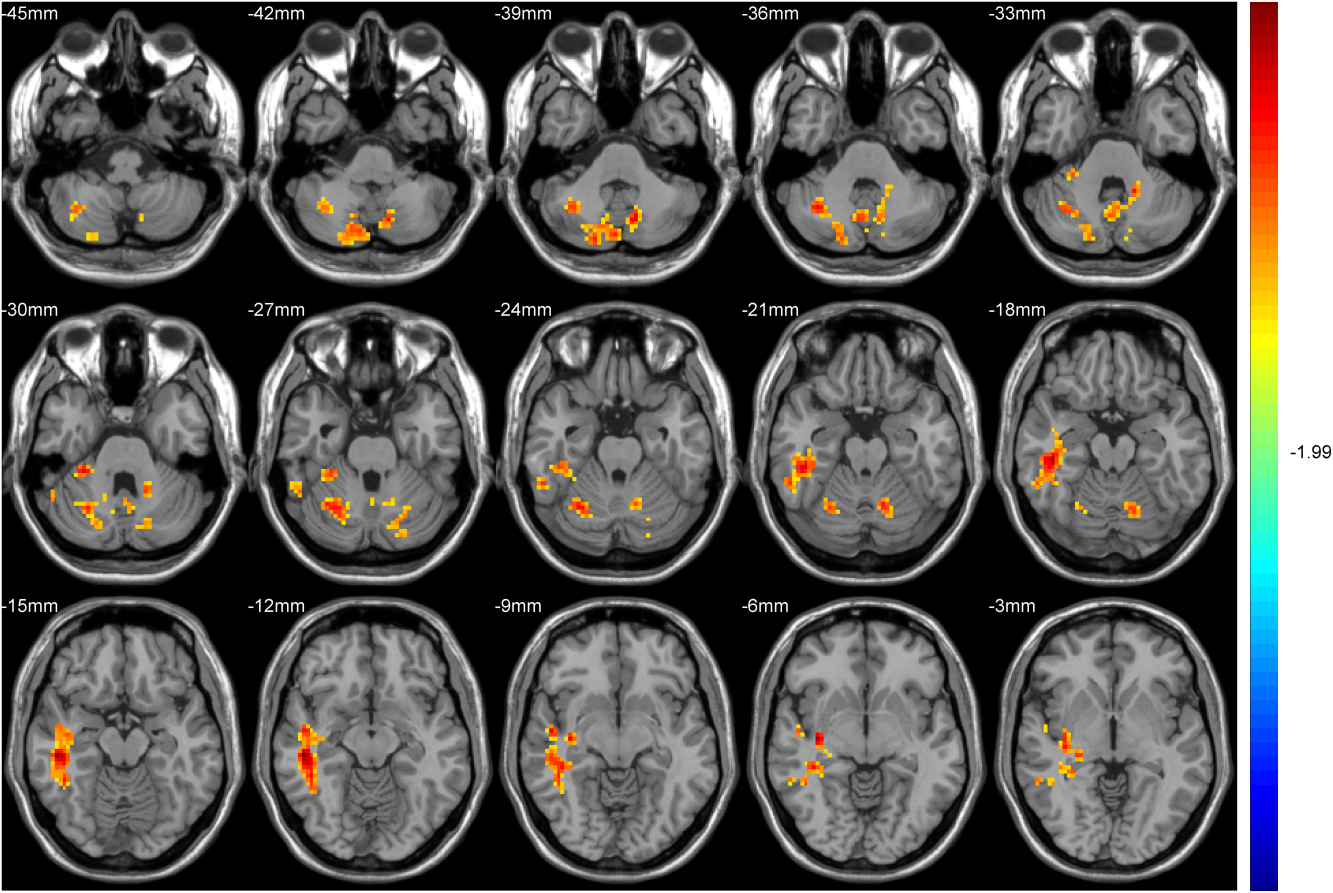
The colored brain regions represent significantly increased functional connectivity (FC) in type 2 diabetes mellitus (T2DM) patients compared with healthy controls (HCs). The color bar indicates the *t*-value from two-sample *t*-tests.

Hub-based FC analysis showed the brain regions with increased FC in the T2DM group mainly involving the right cerebellum crus II, right cerebellum lobule VI, right temporal inferior gyrus, right temporal middle gyrus, and right hippocampus (Table 3, Figure 2).

**Table 3:** Brain regions with increased FC in T2DM compared with HCs.

**Cluster**	**Brain regions**	**Peak MNI**			**Number of voxels**	***t*-value**
		**X**	**Y**	**Z**		
Cluster 1	Right cerebellum crus II Right Cerebellum lobule VI	−12	−69	−39	404	3.6218
Cluster 2	Right temporal inferior gyrus Right temporal middle gyrus Right Hippocampus	51	−27	−12	477	4.0098

*FC, functional connectivity; GRF correction (P < 0.01, voxel P < 0.01, cluster P < 0.05)*.

### Correlation Analysis Results

**Figure 3:**
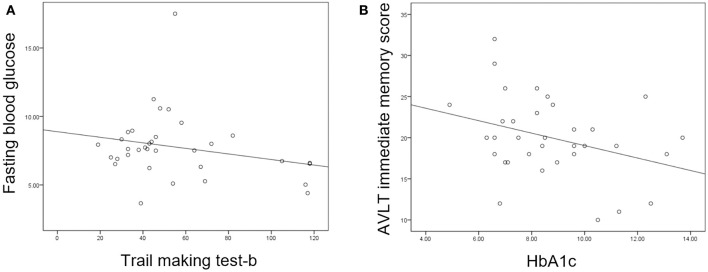
**(A)** Fasting blood glucose was negatively correlated with trail making test-B score (*P* = 0.047, *r* = −0.360). **(B)** Glycosylated hemoglobin (HbA1c) was negatively correlated with auditory verbal learning test (AVLT) immediate recall score (*P* = 0.021, *r* = −0.413).

Partial correlation analysis found that HbA1c was negatively correlated with AVLT immediate memory scores in the T2DM group and FBG was negatively correlated with TMT-B (Figure 3). However, there were no correlations between increased DC or FC and scores of the neuropsychological tests.

## Discussion

This study focused on changes of brain functional hubs and their connectivity in T2DM without MCI by using DC and FC. Compared with the HCs, the T2DM subjects exhibited significantly increased DC in the right cerebellum regions III–V. And according to the hub-based analysis, we found that FC increased between the right cerebellum regions III–V and the right cerebellum crus II/VI, right inferior/middle temporal gyrus, and right hippocampus in the T2DM group. The increased DC and FC may provide evidence of early compensatory mechanism in T2DM without MCI.

### Cognition Assessment of Type 2 Diabetes Mellitus and Healthy Controls

There was no significant difference of neuropsychological assessment between T2DM and HCs, except for a few scales, i.e., MoCA-B, CDT, and SDT. Most of the T2DM subjects were younger than 50 years and had no obvious vascular complications, such as retinopathy and cerebrovascular diseases. This may explain why cognitive functions of the T2DM group are not significantly impaired (5). MoCA-B mainly assesses attention and concentration and has different sensitivity in different mental disorder diseases (35). Longer test durations of CDT and SDT were commonly reported in T2DM patients compared with HCs, indicating that mild impairment of executive function and inflexibility of movement firstly exist at the early stage of the disease (36, 37). Negative correlation between cognitive function and clinical measurement (i.e., HbA1c and FBG) suggested that higher blood sugar level may have some impact on cognitive decline in preclinical stage (38).

### Degree Centrality Alteration in Cerebellum Regions in Type 2 Diabetes Mellitus

DC quantifies local degree, which measures the centrality of every voxel in the human brain connectome (22, 24). The increased DC may imply the transition from an economic connection to the costly connection; that is, brain regions with high energy-consumption such as brain hubs can be reconfigured to meet different and variable cognitive needs through negotiations between connection costs and the topological characteristics of the networks (39, 40). In the current study, increased DC of the right cerebellum regions III–V in T2DM compared with HCs could be attributed to a compensatory mechanism invoked following dysfunction of the neuro architectural network that typically would support a cognitive task (41, 42). The compensation mechanisms of the cerebellum in neural functions in T2DM individuals with the early stage had been reported via not only rs-fMRI but also task fMRI or structure MRI studies (42–45). Our previous study also indicated that T2DM subjects exhibited increased nodal efficiency in the right cerebellum III, which were associated with cognitive performance (41). However, some previous studies reported that T2DM subjects showed significant decreased anatomical connections, mean regional homogeneity (mReHo), cerebral blood flow (CBF), and DC in cerebellar regions (46–48), providing evidence to the significant role of the cerebellum in terms of cognitive function. Although inconsistent results were obtained, it is believed that the cerebellum might play an important role in maintaining normal cognitive functions in T2DM before the onset of symptoms or even at the prediabetic stage (15, 43).

### Functional Connectivity Alteration in Type 2 Diabetes Mellitus

The FCs are accumulation of the paired regional connections in the brain and are rich in information associated with the intrinsic interactions among the brain regions, which are caused by the spontaneous neural activities. Higher FCs indicate the enhancement of temporal consistency of the different brain regions. In the present study, increased FC based on the hub DC analysis was found not only within different cerebellar regions but also between ipsilateral cerebrocerebellar regions, suggesting sophisticated recruitment capacity of the cerebellum in T2DM (15, 42, 43). Though the cognitive functions of the cerebellum are not yet well-understood, a study based on exceptionally large and high-quality dataset analysis indicated that functional gradients of the cerebellum correspond to intrinsic connectivity of cerebellar voxels with the rest of the cerebellum (49). On the other hand, correlation studies have shown interactions between the cerebellum and non-motor areas of the cerebral cortex (50). The inferior and middle temporal lobes contain fiber tracks (e.g., middle cerebellar peduncle and unciform fasciculus) crossing several brain regions and associating with language processing, verbal working memory, visual spatial perception (51), and so forth. The hippocampus is involved in different aspects of memory formation and learning process (10) and plays an important role during the cognitive process of normal cognition to Alzheimer's disease. It was reported that T2DM subjects usually has existing atrophy of the hippocampus and reduced cortical thickness in the right middle and inferior temporal gyri and at the early stage of the disease (12, 52). The increased FC between the cerebellum and temporal gyrus (including the hippocampus) might present the potential impairment of cognition, which could be compensated by means of adjustment of functions of other regions to “recover” the potential impairments before the clinical onset of cognitive impairment.

We did not find any correlation between the aberrant FC and the multiple neurocognitive assessment scale scores. This is probably because the subjects' cognitive function was still in the normal range, and it was not linear enough with the increased DC or FC brain regions. In addition, in most of the cases, correlations could exist in any area, even that without group difference. Even further searching correlations in an area with group difference itself do not guarantee that only patients show a correlation but controls do not. Therefore, we feel that it is more reasonable to do exactly the same exhausted search, for group difference detection, as well as for correlation identification, based on the exploratory nature of this work (53).

### Limitations

There were some limitations in this study. Firstly, it was a cross-sectional investigation and had relatively small sample size. Further longitudinal studies are needed to elaborate the changes in the whole brain DC associated with cognitive impairment, particularly as it develops to MCI or AD. Secondly, uncontrolled thoughts and/or other activities during the resting-state scan could confound the result (43, 54). In addition, different doses of hypoglycemic agents may affect the results, though the blood sugar level of all the T2DM individuals was well-controlled, and we have tried to control for confounding factors. Future studies should take these into consideration, and multimodal MRI that provide comprehensive channels of information should be conducted (55).

## Conclusion

The present study found increased DC in the right cerebellum regions III–V, as well as increased FCs within cerebellar regions and ipsilateral cerebrocerebellar regions, indicating complicated compensatory mechanisms of the cerebellum in the early stage of T2DM without MCI. These results provide valuable insights into the neurological pathophysiology of T2DM-related of cognitive decline and hold great potential in detecting early imaging markers of T2DM.

## Data Availability Statement

The raw data supporting the conclusions of this article will be made available by the authors, without undue reservation.

## Ethics Statement

The studies involving human participants were reviewed and approved by Medical Research Ethics Committee of Guangzhou University of Chinese Medicine. The patients/participants provided their written informed consent to participate in this study.

## Author Contributions

YLi and YLia designed the entire experiment and completed the data analysis and manuscript writing. XT analyzed the data and revised the manuscript. YC has made contributions to statistical analysis. JY administered the neuropsychological tests. HZ and CQ contributed to the review of conventional sequence images. YF and XM contributed to data collection. SQ is the guarantor of this study and had complete access to all data in the study. All the authors accept responsibility for the integrity of the data and the accuracy of the data analysis.

## Conflict of Interest

The authors declare that the research was conducted in the absence of any commercial or financial relationships that could be construed as a potential conflict of interest.
