# Supplementary figures and images for: Altered Functional Hubs and Connectivity in Type 2 Diabetes Mellitus Without Mild Cognitive Impairment
Source: Front Neurol. 2020 Sep 11;11:1016. doi: 10.3389/fneur.2020.01016 (PMC7533640; doi:10.3389/fneur.2020.01016)

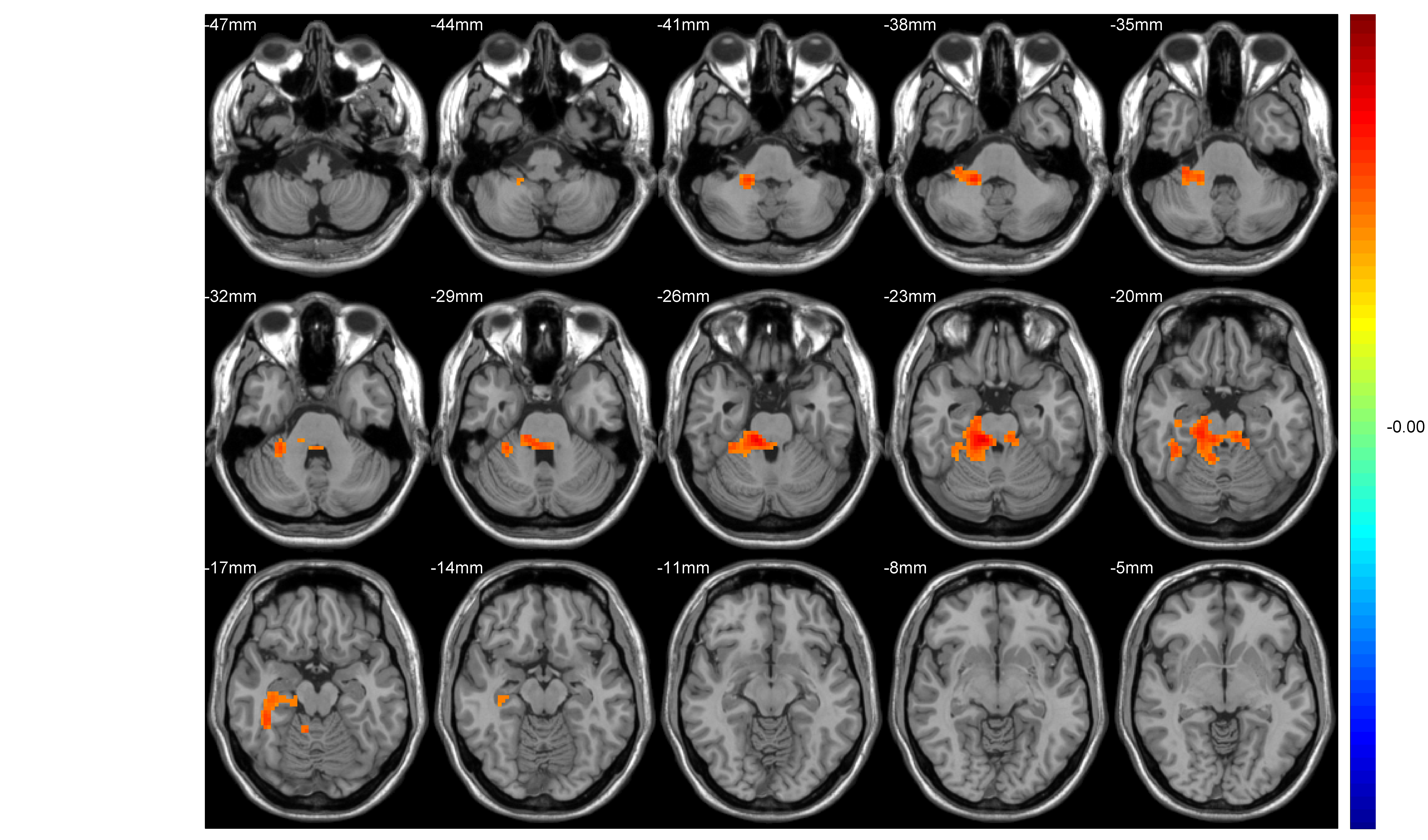

Supplement: Supplementary file 1 [file Image_1.tiff]

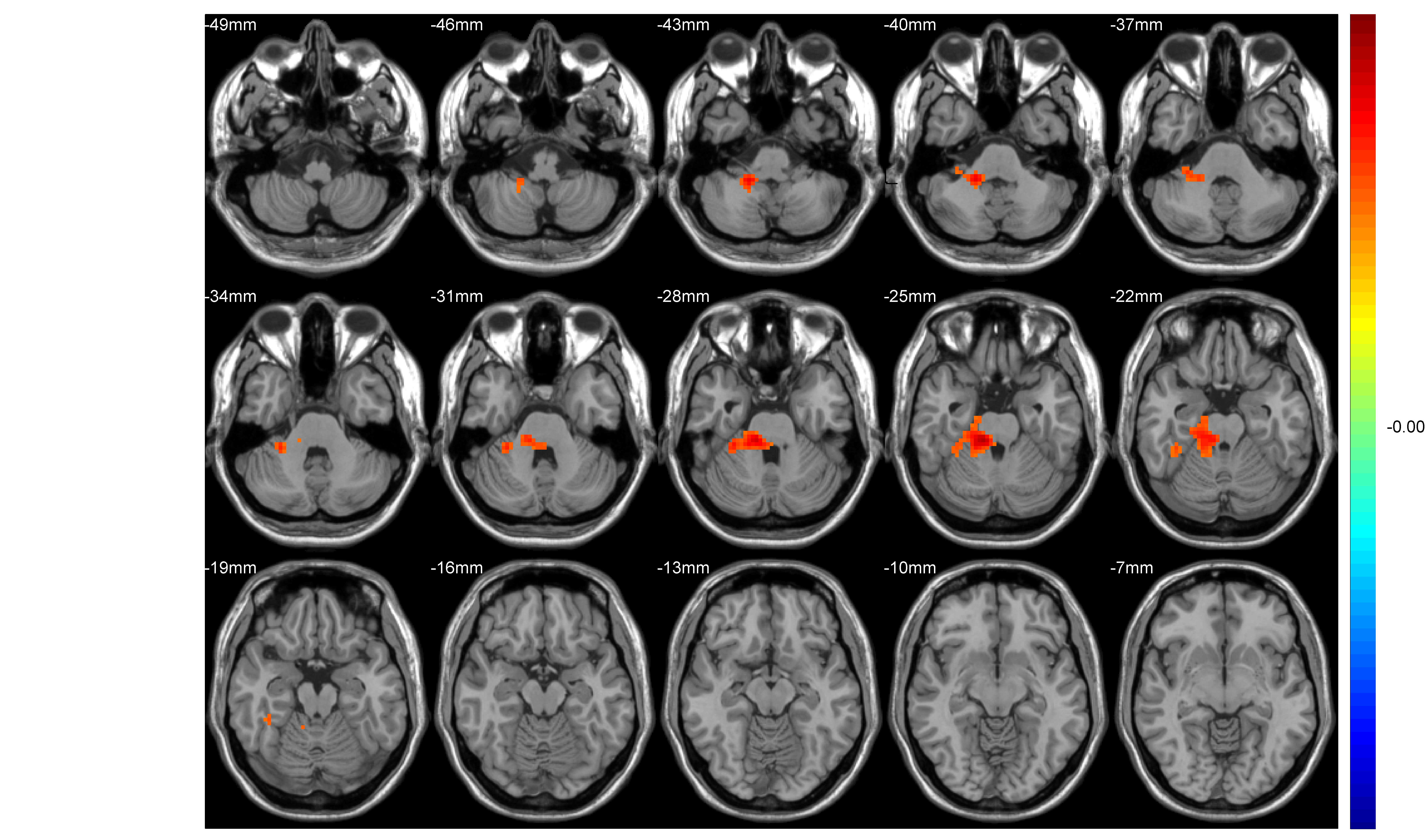

Supplement: Supplementary file 2 [file Image_2.tiff]
